# Psychometric Properties of the Preference for Intuition and Deliberation in Eating Decision-Making Scale among Brazilian Adult Women

**DOI:** 10.3390/nu16193252

**Published:** 2024-09-26

**Authors:** Thainá Richelli Oliveira Resende, Edilene Márcia de Sousa, Marle dos Santos Alvarenga, Mariana Cristina Palermo Ferreira, Larissa Stefhanne Damasceno de Amorim Póvoa, Leandro Henrique Pereira Galvane, Cleidiel Aparecido Araujo Lemos, António Raposo, Ariana Saraiva, Conrado Carrascosa, Hmidan A. Alturki, Pedro Henrique Berbert de Carvalho

**Affiliations:** 1 Body Image and Eating Disorders Research Group (NICTA), Federal University of Juiz de Fora, Governador Valadares 35010-180, MG, Brazil; thaina.richelli@gmail.com (T.R.O.R.); edilenemarcia@yahoo.com.br (E.M.d.S.); mariana.palermo@ufjf.br (M.C.P.F.); 2 Postgraduate Program in Applied Health Sciences (PPgCAS), Federal University of Juiz de Fora, Governador Valadares 35010-180, MG, Brazil; dralarissapovoa@gmail.com (L.S.D.d.A.P.); leandro.galvane@hotmail.com (L.H.P.G.); 3 Department of Nutrition, School of Public Health, University of São Paulo, São Paulo 01246-904, SP, Brazil; marlealvarenga@gmail.com; 4 Department of Dentistry, Federal University of Juiz de Fora, Governador Valadares 35010-180, MG, Brazil; cleidiel.lemos@ufjf.br; 5 CBIOS (Research Center for Biosciences and Health Technologies), Universidade Lusófona de Humanidades e Tecnologias, Campo Grande 376, 1749-024 Lisboa, Portugal; 6 Department of Animal Pathology and Production, Bromatology and Food Technology, Faculty of Veterinary, Universidad de Las Palmas de Gran Canaria, Trasmontaña s/n, 35413 Arucas, Spain; ariana_23@outlook.pt (A.S.); conrado.carrascosa@ulpgc.es (C.C.); 7 King Abdulaziz City for Science & Technology, Wellness and Preventive Medicine Institute—Health Sector, Riyadh 11442, Saudi Arabia; 8 Eating Disorders Program, Institute of Psychiatry (AMBULIM), University of São Paulo, São Paulo 05403-010, SP, Brazil

**Keywords:** cross-cultural adaptation, decision-making, feeding behaviors, intuitive eating, measurement, psychometrics, reliability, validity

## Abstract

The Preference for Intuition and Deliberation in Food Decision-Making Scale (E-PID) was developed to evaluate both intuitive and deliberative food decision-making within a single instrument. However, its psychometric properties have only been assessed among German-speaking participants. The main aim of the present study was to evaluate evidence of validity and reliability of the E-PID among 604 Brazilian adult women. Exploratory (*n* = 289) and confirmatory factor analyses (*n* = 315) were conducted to evaluate the factor structure of the E-PID. Convergent validity was assessed correlating the E-PID with measures of eating behaviors (Tree-Factor Eating Questionnaire-18), intuitive eating (Intuitive Eating Scale-2), and a measure of beliefs and attitudes towards food (Food-Life Questionnaire-SF). McDonald’s Omega coefficient (ω) was used to test the internal consistency of the E-PID. Results from an exploratory and confirmatory factor analysis supported a two-factor structure with seven items. We found good internal consistency (McDonald’s *ω* = 0.77–0.81). Furthermore, the E-PID demonstrated adequate convergent validity with measures of intuitive, restrictive, emotional and uncontrolled eating, and beliefs and attitudes towards food. Results support the use of the E-PID as a measure of intuition and deliberation in food decision-making among Brazilian adult women, expanding the literature on eating decision-making styles.

## 1. Introduction

Decision-making related to eating is a complex behavior influenced by factors beyond the human body’s physiological need to maintain homeostasis. Sociocultural, environmental, and individual factors play significant roles in choices concerning how, where, what, when, and how much to eat [1,2,3]. It is noteworthy that although it has its principle in the primitive and automatic instinct of “I need to look for food to stay alive”, psychological aspects, including mental health status, distractions, external cues, and social influences, often dominate eating decisions [1].

The sociocultural factors heavily mediate eating behavior and can elucidate individual motivation for food choices. Theoretical models such as the Dual-Pathway [4], Social-Cognitive [5], and Social Comparison [6], when analyzed from the perspective of eating decision-making, support the idea that cultural environments exert significant influence on individuals’ choices, reflecting social norms and values. According to Higgs [2], social influences on food are powerful and widespread. Individuals have a highly developed ability to learn from others and find their approval rewarding and their disapproval aversive. Consequently, social rules can affect food choices and consumption patterns, affecting self-perception and the sensorial/hedonic evaluation of food [2].

In view of the above, decision-making related to eating is not solely a natural and instinctive behavior but is also influenced by environmental factors. Pachur and Spaar [7] and König et al. [8] have reported that individuals may employ either spontaneous (i.e., intuitive) and/or effortful/planned (i.e., deliberative) decision-making modes when making food-related choices. Depending on this decision, eating behavior outcomes can be reinforced [9]. Recent studies suggest that individuals who decide to eat intuitively tend to have a better relationship with food [10,11,12]. Intuitive eating is defined as being connected to internal hunger, satiety, and appetitive cues, and flexibly using these cues to determine when, what, and how much to eat [12].

Social-cognitive models of health behavior and social-cognitive concepts such as self-efficacy reflect a deliberate and controlled approach to eating [8]. According to these models, planning and goal-setting are thought to be predictive of healthy eating and related to health outcomes [8]. However, individuals who follow external deliberation for their decision-making, often characterized by conscious efforts to reduce food intake, use food to meet emotional needs, and rigorous control over eating may paradoxically lead them to consume larger quantities of food [8].

Several measures have been developed to comprehensively assess different eating behaviors. Some instruments focus on external factors influencing eating behavior motivation when eating, such as the Three-Factor Eating Questionnaire [13] and the Dutch Eating Behavior Questionnaire [14], while others are focused on assessing eating according to behaviors aligned with physiological signs of hunger and satiety cues, such as the Intuitive Eating Scale-2 [15,16].

In 2021, König et al. [8] developed a scale, the Preference for Intuition and Deliberation in Eating Decision-making (E-PID), designed to assess individuals’ tendencies towards intuitive versus deliberative decision-making in the context of eating. The authors evaluated the E-PID psychometric properties in a sample of 699 German participants, predominantly women (79.69%), with a mean age of 28.59 years (SD = 11.43). Using confirmatory factor analysis (CFA), the authors confirmed the two-factor structure for the E-PID, comprising seven items. Furthermore, the E-PID subscales showed good reliability (Cronbach’s *α* = 0.78—Preference for intuition; and *α* = 0.81—Preference for deliberation). Moreover, evidence of convergent validity was established through correlations between the E-PID subscales and measures of intuitive eating and restrictive eating [8]. A positive correlation was found between decision-making through intuition and intuitive eating (*r* = 0.31; *p* < 0.001), indicating that individuals with a high preference for intuition are more likely to decide their food choices on internal cues, such as hunger and satiety signals [8]. Conversely, individuals with a high preference for deliberation tend to rely on cognitive regulation of eating, as demonstrated by a high correlation with restrictive eating (*r* = 0.52; *p* < 0.001). König et al. [8] concluded that these two eating decision-making styles (i.e., preference for intuition and preference for deliberation) are distinct from each other.

Although one might argue that the E-PID evaluates similar constructs as other established scales, such as the Dutch Eating Behavior Questionnaire [14] and the Intuitive Eating Scale-2 [15,16], this assumption may not hold. The E-PID assesses both intuitive and deliberative decision-making in eating, which is not captured in other scale measures. Evidence from König et al. [8] supports this assertion, as the correlation between the Preference for intuition subscale and the Intuitive Eating Scale-2, and the Preference for the deliberation subscale and restrictive eating was only moderate [8]. Therefore, it is evident that the E-PID offers a novel perspective on eating decision-making that complements existing measures in the field [8].

Instruments for research on diverse cultural populations must be comparable among groups and sensitive to contextual differences in the local area [17]. In other words, when an instrument is translated and utilized in a new language, researchers must ensure that the translated measure is consistent with the original measure and that the instrument applies to various groups in a similar way [17]. Given that psychometric properties of an instrument should be evaluated across different nationalities, and considering the originality of the E-PID in assessing intuitive and deliberative use in eating decision-making styles combined into a single measure, the present research aimed (1) to evaluate the factor structure of the E-PID through using an exploratory (EFA) and CFA factor analytic approach; (2) to examine evidence of convergent validity of the E-PID with measures of intuitive, emotional, restrictive, and uncontrolled eating, as well as beliefs and attitudes towards food; (3) to estimate the reliability (i.e., internal consistency) of the E-PID among Brazilian adult women.

We hypothesized (Hypothesis 1) that the E-PID would replicate the original two-factor structure designed by König et al. [8] when applied to Brazilian adult women. Further, it was also expected that the Preference for intuition subscale would demonstrate positive correlations with measures of intuitive eating and eating pleasure, while the Preference for deliberation subscale would positively correlate with measures of restrictive, uncontrolled, and emotional eating, and measures of negative beliefs and attitudes towards food (Hypothesis 2). Finally, it was expected to find good internal consistency in the E-PID among Brazilian adult women (Hypothesis 3).

## 2. Materials and Methods

### 2.1. Participants

The current study was part of a cross-sectional investigation carried out in Brazil that aimed to evaluate food choices, eating decision-making processes, and eating behaviors and attitudes among adult women. The current research involved the E-PID’s cross-cultural adaptation and psychometric evaluation [17]. A total of 604 Brazilian adult women with a median age of 26 years (ranged from 18 to 35 years) took part in this study. The sample size exceeded the requirement for EFA and CFA, which typically necessitates 20 participants per item [17]. Hence, for the 7-item scale, a minimum sample size of 140 participants is recommended. The inclusion criteria were Brazilian citizenship, aged over 18 years, and self-identifying as women. Exclusion criteria were the presence of any medical condition capable of directly influencing eating behaviors, such as gastrointestinal disorders (information obtained through self-reporting of the presence of previous diagnosed diseases).

### 2.2. Procedures

All procedures adhered to the principles outlined in the Declaration of Helsinki, and ethical approval was obtained from the relevant Institutional Review Board (approval number 5.869.779).

Participants were enlisted via advertisements on social media platforms (Instagram^®^, Facebook^®^, WhatsApp^®^, and Telegram^®^) and online communities. Additionally, requests for collaboration in spreading the research were extended to higher education institutions via email. To enhance the dissemination of the research, posters containing QR codes were displayed in healthcare facilities catering to women. These QR codes directed participants to a hyperlink, allowing them to access the full study’s protocol via Google Forms^®^. All individuals participated voluntarily without any remuneration or subsidy.

### 2.3. Measures

#### 2.3.1. Demographic Data

Participants self-reported their (a) age, (b) sex assigned at birth, (c) gender identity, (d) sexual orientation, (e) income, (f) race/ethnicity, (g) body mass, and (f) height. Using the formula recommended by the World Health Organization [18], we calculated the body mass index (BMI). The following official race/ethnicity categories from the Brazilian census [19] were used: White, Brown, Black, and Other. Brazilian economic classification criteria [20] were used, and income categories were high, mid, and low.

#### 2.3.2. Decision-Making Related to Eating

Decision-making related to eating, either through intuition or deliberation, was assessed using the E-PID [8]. König et al. [8] found a two-factor solution with 7 items for the E-PID: *Preference for intuition* and *Preference for deliberation*. The items are graded on a five-point Likert-type scale (1 = completely disagree to 5 = agree) so that greater scores denote a greater preference for intuition or deliberation. The two subscales had adequate internal consistency: preference for intuition (Cronbach’s *α* = 0.79) and preference for deliberation (*α* = 0.82) [8]. Using the guideline from Swami and Barron [17], we cross-culturally adapted the English version of the E-PID to Brazilian Portuguese to be able to use the measure in the present study (please see Appendix A).

#### 2.3.3. Eating Behaviors

Distinct eating behaviors were measured though the Three-Factor Eating Questionnaire-18 [13]. The TFEQ-18 is composed of 18 items divided into three subscales [13]: *Cognitive restrictive* (CR), *Emotional eating* (EE), and *Uncontrolled eating* (UE). It is answered on diverse Likert-type scales and the total score varies from 18 to 76. A higher score indicates a greater likelihood of disordered eating behaviors [13]. The reliability of the instrument was considered adequate (composite reliability = 0.87–0.89; *α* = 0.86–0.89) [13]. The validated Brazilian version of the TFEQ-18 was applied [13]. We calculated the inter-item correlation (i.e., Spearman’s correlation) to estimate the inter-item reliability of the CR subscale (*rho* = 0.209–0.772; *ps* < 0.001). The internal consistency of the TFEQ-18 was good for both the UE subscale (*ω* = 0.820; 95% confidence interval [CI] = 0.80–0.84]) and the EE subscale (*ω* = 0.90; 95% CI = 0.89–0.92).

#### 2.3.4. Intuitive Eating

Intuitive eating was evaluated through the IES-2 [16]. The IES-2 is composed of 23 items divided into four subscales: UPE—*Unconditional permission to eat*, EPRER—*Eating for physical rather than emotional reasons*, RHSC—*Reliance on hunger and satiety* cues, and BFCC—*Body–food choice congruence*. The IES-2 items are rated on a five-point Likert-type scale (1 = never to 5 = always), so that higher scores indicate a greater likelihood of intuitive eating. All subscales showed adequate internal consistency (*α* = 0.79–0.89) [16]. The validated Brazilian version of the IES-2 was used in the present study [16]. We found good internal consistency of the IES-2 total score and its subscales (total score—*ω* = 0.90; 95% CI = 0.89–0.92; UPE—*ω* = 0.68; 95 CI% = 0.64–0.72; EPRER—*ω* = 0.89; 95 IC% = 0.88–0.91; RHSC—*ω* = 0.91; 95 IC% = 0.90–0.92; and BFCC—*ω* = 0.91; 95 IC% = 0.90–0.92).

#### 2.3.5. Beliefs and Attitudes Toward Food

Beliefs and attitudes toward food were measured through the FLQ-SF [21]. The FLQ-SF is composed of 22 items divided into four subscales: *Weight concern* (WC), *Diet–health orientation* (DHO), *Belief in a diet–health linkage* (DHL), and *Food and pleasure* (FP). The FLQ-SF items are rated on a seven-point Likert-type scale (1 = completely disagree to 7 = completely agree) so that greater scores indicate a higher emphasis in each subscale [21]. We applied the Brazilian version of the FLQ-SF [22], composed of 17 of the 22 original items and the original four subscales [22]. All subscales demonstrated adequate internal consistency: WC (*ω* = 0.80; 95% CI = 0.77–0.84), DHL (*ω* = 0.89; 95% CI = 0.87–0.91), FP (*ω* = 0.79; 95% CI = 0.75–0.83), and DHO (*rho* = 0.36; 95% CI = 0.28–0.42) [21].

### 2.4. Statistics

#### 2.4.1. Descriptive Data Analyses

Item-level missing data were inspected (Little’s MCAR test; *p >* 0.05), found to be consistent with missing completely at random, and were replaced using the expectation–maximization method [23]. The final sample was composed of 594 Brazilian women and was divided randomly into two subsets: one for EFA (*n* = 289) and another for CFA (*n* = 315). Items in the Brazilian version of the E-PID were inspected for univariate and multivariate normality, using the skewness (*Sk* < 3) and kurtosis (*Ku* < 7) and Mardia’s coefficients (<5), respectively. We inspected multivariate outliers (Mahalanobis distance; *D*^2^). Similarity between EFA and CFA samples were tested using either the Mann-Whitney *U* test for independent samples or the chi-squared test (*χ*^2^) of association.

#### 2.4.2. Factor Analysis

To explore the E-PID factor structure, we applied an EFA using principal-axis factoring and oblique rotation (i.e., oblimin). The Bartlett’s sphericity test (*p* < 0.05) and Kaiser–Meyer–Olkin test (KMO > 0.70) were used as measures of sampling adequacy. Parallel analysis was used to evaluate the number of factors and items to be retained [17]. Items with a factor loading (*λ*) ≥ 0.60 were retained [17].

We ran a CFA using weighted least squares means and variance adjusted (WLSMV) to test the factor solution of the E-PID identified previously through EFA. Acceptable model fit was evaluated based on the chi-square test weighted by degrees of freedom (*χ*^2^/*df* < 3), and several fit indices: the comparative fit index (CFI close to 0.95), Tucker–Lewis’s index (TLI close to 0.95), root mean square error of approximation (RMSEA below 0.08; 90% confidence interval [CI]; *p* > 0.05), and standardized root mean square residual (SRMR below 0.08) [24]. Modification indices (MI) were examined for values > 3.84 [25].

#### 2.4.3. Convergent Validity

Evidence of convergent validity was examined via *rho* between the E-PID *Preference for intuition* subscale and measures of intuitive eating (i.e., the IES-2 total scores and its subscales), and eating pleasure (i.e., the FLQ-FP subscale). Furthermore, there were correlations between the E-PID *Preference for deliberation* subscale and measures of restrictive, emotional, and uncontrolled eating (i.e., the TFEQ-UU, TFEQ-EE, and TFEQ-CR subscales) and measures of weight concern (i.e., the FLQ-WC subscale), diet–health orientation (i.e., the FLQ-DHO subscale), and beliefs in diet–health linkages (i.e., the FLQ-DHL subscale). Based on Cohen’s recommendation, correlations ~0.10 were small/weak, correlations ~0.30 were medium/moderate, and correlations >0.50 were large/strong [26].

#### 2.4.4. Internal Consistency

McDonald’s omega (*ω*) coefficient was used to estimate the internal consistency of all applied measures. Values > 0.70 were considered adequate [27].

## 3. Results

### 3.1. Descriptive Statistics

The EFA and CFA sample characteristics are shown in Table 1. The EFA and CFA samples did not differ in any demographic data (*ps* > 0.05).

### 3.2. Factor Analysis

No multivariate outliers were identified. The KMO was 0.73, and Bartlett’s test of sphericity was significant (*χ*^2^ [21.000] = 710.987; *p* < 0.001). Model fit was good: CFI = 0.98; TLI = 0.96; RMSEA = 0.06 (90% CI = 0.01–0.10; *p* > 0.05); and SRMR = 0.02. The parallel analysis showed that a two-factor structure was the most appropriate (see Supplementary Materials Figure S1). All factor loadings (*λ*) were > 0.61 (please see Table 2).

The two-factor solution of the E-PID was confirmed through CFA, showing a good fit to the data: *χ*^2^/*df* = 2.61; CFI = 0.97; TLI = 0.96; RMSEA = 0.072 (90% CI = 0.43–0.10; *p* > 0.05); and SRMR = 0.67. Standardized factor-loading estimates for the E-PID were all adequate (Table 3). Furthermore, it was decided not to use the modification indices.

### 3.3. Convergent Validity

The *Preference for intuition* subscale demonstrated a positive and large correlation with IES-RHSC, a positive and medium correlation with the IES-2 total score and the IES-UPE; and a positive and small correlation with the IES-EPRER, IES-BFCC, and FLQ-FP. The *Preference for deliberation* subscale demonstrated a positive and large correlation with the FLQ-DHO, and a positive, small correlation with the FLQ-WC, FLQ-DHL, TFEQ-EU, and TFEQ-EE (Table 4).

### 3.4. Internal Consistency

The internal consistency of the E-PID subscales was good: *Preference for intuition* (*ω* = 0.77; 95% CI = 0.74–0.80) and *Preference for deliberation* (*ω* = 0.82; 95% CI = 0.80–0.84).

## 4. Discussion

In line with the necessity to assess the complexity of decision-making regarding food choices using suitable instruments, this study evaluated the psychometric properties of the E-PID among Brazilian adult women. The results support construct and convergent validity, and reliability through internal consistency of the E-PID among Brazilian adult women.

The results from the EFA and CFA confirmed the first hypothesis: the original two-factor solution of the E-PID (i.e., *Preference for intuition* and *Preference for deliberation* in eating decision-making) with seven items, as proposed by König et al. [8], showed good fit indices. Although this is the first study to validate the factorial structure of the E-PID in a new cultural context, the factor structure of the instrument appears robust, as evidenced by good factor loadings and the adjustment fit indices. The present study used a two-step analytic approach (i.e., EFA-to-CFA), which should be considered an advance, given that König et al. [8] evaluated the factor structure of the E-PID applying only CFA. According to Swami and Barron [17], analyzing the fit of competing models that have been put out in the translational literature is also helpful. One should think about whether or not all suggested paths make sense theoretically or if some should be included because of pertinent theory [17].

Confirming the second hypothesis, the *Preference for intuition* and the *Preference for deliberation* subscales demonstrated distinct associations with convergent measures, suggesting that preferences for intuitive and deliberative approaches to making eating decisions may represent two distinct decision-making styles. Specifically, the *Preference for intuition* subscale showed a positive correlation with constructs such as intuitive eating, unconditional permission to eat, eating for physical rather than emotional eating, congruence of the choice of food for body functionality, and eating pleasure. König et al. [8] also found that the *Preference for intuition* subscale was positively correlated with intuitive eating. Intuitive eating is a construct that has as its premise respect and trust in the body’s internal signals to determine the time to eat and the amount needed. Intuitive eaters typically experience harmony between feelings of hunger, satiety, and food satisfaction, possess body awareness, prioritize bodily cues over external cues, and reject a dieting mentality [12,28]. Our results extend the findings of König et al. [8] by demonstrating correlations between the *Preference for intuition* subscale and all subscales of the IES-2.

Moreover, the correlation between the intuitive eating-decision styles with eating pleasure was also observed. The statement “I think about food in a positive way” from the *Food and pleasure* subscale from FLQ-SF aligns with the goal of intuitive eating, which aims to help people improve their relationship with food by emphasizing respect for internal signals. A positive relationship with food encompasses more than just making choices based on nutritional needs, hunger, and satiety; it must also respect the pleasure of eating. This is considered an important focus even for motivation for healthy eating [12,28,29].

The *Preference for deliberation* subscale showed a positive correlation with weight concerns, diet–health orientation, and beliefs in a diet–health linkage. These results are consistent with the findings of König et al. [8]. The authors found that a deliberative eating decision-making style was associated with eating healthily, engaging in healthy eating behavior, and considering health-related parameters. Our results from the convergent validity analysis also demonstrated that the *Preference for deliberation* subscale was correlated with higher restrictive, emotional, and uncontrolled eating. König et al. [8] also found that a higher preference for deliberation was positively related to restrained eating. Individuals who control their eating in a restrictive way may have difficulty maintaining the homeostatic system of hunger and satiety, activating ‘hedonic’ reward pathways associated with the palatability (e.g., sight, smell, and taste) of food, making them eat according to external motivations, such as emotional ones [30,31,32].

Confirming the third hypothesis, an adequate internal consistency for both E-PID subscales was observed, with results in line with those found in the original study [8]. It is highlighted that König et al. [8] used the Cronbach alpha coefficient (*α*) to estimate the internal consistency of the E-PID, while we used the McDonald’s omega coefficient (*ω*). The use of McDonald’s *ω* has been recommended over Cronbach’s *α* [33].

Based on our findings, we consider that food choices based on intuition and relying on body signals to predict how much, how, and what to eat can be healthier for mental and social health. In contrast, food choices based on external signs (i.e., deliberation), often influenced by social norms, can harm the physical and psychological health of individuals. Thus, health professionals may promote gentler nutrition and help their patients to use intuition during eating decision-making. The strengths of the present study include the following: (a) validating a measure of decision-making regarding food, considering both intuition and deliberation within a new sociocultural context; (b) incorporating measures of restrictive, emotional, and uncontrolled eating measures, which have not been used in the previous validation of the E-PID [8]; (c) meeting the literature criteria for an adequate number of subjects for validation studies [17]; and (d) use of robust psychometric analyses for factor analysis [17].

However, several limitations should also be noted: (a) Generalizability of the results found in the present study to all Brazilian adult women is limited due to the use of a non-probabilistic sample. (b) Use of self-reported measures might have introduced a social desirability bias among participants. (c) Recruitment through social media and networks might have led to sample overrepresentation. Due to Brazil’s continental dimensions, it would be extremely expensive and time-consuming to collect data in paper-and-pencil format in different regions, so future research could conduct a multicenter study to explore its advantages and disadvantages in relation to online data collection. (d) Other evidences of validity (e.g., discriminant validity) and reliability (e.g., temporal stability) of the E-PID were not investigated. (e) We exclusively included young women; future studies should explore the psychometric properties of E-PID in Brazilian men. (f) The health conditions and lifestyle of the population were not considered. Therefore, future studies should address these concerns.

## 5. Conclusions

In summary, the findings indicate that the E-PID replicates its original two-factor structure. Furthermore, the *Preference for intuition* subscale correlates positively with measures of intuitive eating and eating pleasure, while the *Preference for deliberation* subscale correlates positively with measures of restrictive, emotional, and uncontrolled eating, weight concerns, diet–health orientation, and beliefs in a diet–health linkage. Finally, good internal consistency was found for the Brazilian Portuguese version of the E-PID among Brazilian adult women. Taken together, these results support the use of the E-PID as a measure of intuitive and deliberative use in food decision-making among Brazilian adult women, expanding the literature on eating decision-making styles.

## Figures and Tables

**Table 1:** Descriptive data analyses and test of differences between exploratory and confirmatory factor analysis samples.

Variables	Exploratory Factor Analysis (*n* = 289)	Confirmatory Factor Analysis (*n* = 315)	Statistics ^c^
**Age (years) ^a^**	26 (18–35)	25 (18–35)	*U* = 43,448; *p* = 0.333
**Body mass index (kg/m^2^) ^a^**	23.43 (16.03–46.29)	23.50 (16.03–46.29)	*U* = 46,259; *p* = 0.729
**Race/ethnicity ^b^**			
White	171 (59.17%)	170 (53.96%)	*χ*^2^ (3) = 3.196; *p* = 0.362
Brown	97 (33.56%)	111 (35.24%)	
Black	16 (5.54%)	28 (8.89%)	
Other	5 (1.73%)	6 (1.91%)	
**Gender identity ^b^**			
Cisgender	237 (82.01%)	263 (83.49%)	*χ*^2^ (2) = 0.444; *p* = 0.801
Non-Cisgender	2 (0.69%)	3 (0.95%)	
Prefer not to respond	50 (17.30%)	49 (15.56%)	
**Sexual orientation ^b^**			
Heterosexual	247 (85.46%)	270 (85.72%)	*χ*^2^ (3) = 1.25; *p* = 0.740
Lesbian	4 (1.38%)	8 (2.54%)	
Others	35 (12.12%)	34 (10.79%)	
Prefer not to respond	3 (1.04%)	3 (0.95%)	
**Income ^b^**			
High	212 (73.35%)	232 (73.65%)	*χ*^2^ (3) = 1.64; *p* = 0.438
Mid	62 (21.45%)	64 (20.31%)	
Low	0 (0%)	3 (0.95%)	
Prefer not to respond	15 (5.20%)	16 (5.09%)	

Note: *n* = 604; *p* = *p*-value. Official race/ethnicity categories in the Brazilian Institute of Geography and Statistics (IBGE) [19]. Sexual orientation = others (i.e., asexual, pansexual, bisexual); ^a^ = median (minimum and maximum values); ^b^ = absolute frequency (relative frequency); ^c^ = Mann-Whitney *U* test for independent samples and chi-squared (*χ*^2^) test of association.

**Table 2:** Descriptive data analyses and factor loadings for exploratory factor analysis of the E-PID.

EPID/*Brazilian Portuguese Translation*	*Md* (IQR)	Range	Subscales (*λ*)	
			Deliberation	Intuition
1. When deciding what to eat, I rely on my gut feeling/*Na hora de decidir o que comer, eu confio na minha intuição.*	1 (3)	1–5	−0.01	**0.81**
2. With most eating decisions, it makes sense to completely rely on your instinct/*Na maioria das decisões alimentares, faz sentido confiar totalmente no seu instinto.*	3 (2)	1–5	0.03	**0.82**
3. I am a very intuitive eater/*Eu sou um(a) comedor(a) intuitivo(a).*	3 (2)	1–5	−0.04	**0.65**
4. Before I make eating decisions, I usually think about it/*Antes de tomar decisões alimentares, eu geralmente penso sobre elas.*	4 (2)	1–5	**0.61**	0.17
5. I think more about my plans and goals relating to my eating behaviour than other people/*Eu penso mais nos meus planos e objetivos relacionados ao meu comportamento alimentar do que outras pessoas.*	3 (2)	1–5	**0.72**	−0.03
6. I prefer making plans about my eating behaviour instead of leaving it to chance/*Eu prefiro fazer planos em relação ao meu comportamento alimentar ao invés de deixá-los ao acaso.*	3 (2)	1–5	**0.84**	−0.12
7. I reflect on my eating behaviour/*Eu reflito sobre o meu comportamento alimentar.*	4 (2)	1–5	**0.75**	0.11
Explained variance (subscales)			25.7%	30.7%
Explained variance (total)			56.4%	

Note: *n* = 289; *Md* = median; IQR = interquartile range; *λ* = factor loadings. Values in **bold** indicate that an item is loaded on the corresponding factor (items #1, 2, 3—Preference for intuition subscale; items #4, 5, 6, and 7—Preference for deliberation subscale).

**Table 3:** Confirmatory factor analysis and standardized factor loadings of the respecified model of the E-PID.

					95% CI		
E-PID Subscales	Item	*SE*	z-Value	*p*	Lower	Upper	Standardized *λ*
*Preference for intuition*	1	0.106	9.306	<0.001 *	0.78	1.20	0.80
	2	0.039	21.389	<0.001 *	0.76	0.91	0.84
	3	0.044	14.502	<0.001 *	0.55	0.72	0.64
*Preference for deliberation*	4	0.032	24.732	<0.001 *	0.72	0.85	0.78
	5	0.119	8.024	<0.001 *	0.72	1.19	0.67
	6	0.030	26.310	<0.001 *	0.74	0.85	0.79
	7	0.086	10.272	<0.001 *	0.72	1.06	0.75

Note: *n* = 315; *SE* = standard error; *p* = *p*-value; CI = confidence interval; *λ* = factor loadings. * *p* < 0.001.

**Table 4:** Descriptive data analyses and convergent validity of the E-PID.

Subscales	Range	*Md* (IQR)	*rho*	*p*
*Preference for Intuition*				
IES-2	23–115	75 (24)	0.37	<0.001 **
IES-UPE	6–30	22 (6)	0.36	<0.001 **
IES-EPRER	8–40	23.44 (13)	0.13	0.002 *
IES-RHSC	6–30	19.31 (10)	0.50	<0.001 **
IES-BFCC	3–18	11 (5)	0.17	<0.001 **
FLQ-FP	4–28	36 (14)	0.28	<0.001 **
*Preference for Deliberation*				
FLQ-WC	6–42	23 (13)	0.19	<0.001 **
FLQ-DHO	3–21	14 (6)	0.42	<0.001 **
FLQ-DHL	5–35	32 (7)	0.26	<0.001 **
TFEQ-CR	6–28	15 (2.2)	0.03	0.506
TFEQ-UE	9–36	23 (8)	0.18	<0.001 **
TFEQ-EE	4–16	8 (5)	0.19	<0.001 **

Note: *n* = 604; *Md* = median; IQR = interquartile range; *rho* = Spearman’s rank order correlation coefficient; *p* = *p*-value; IES-2 = Intuitive Eating Scale-2; IES-UPE = Unconditional permission to eat subscale; IES-EPRER = Eating for physical rather than emotional reasons subscale; IES-RHSC = Reliance on hunger and satiety cues subscale; IES-BFCC = Body–food choice congruence subscale; FLQ = Food-Life Questionnaire; FLQ-FP = Food and pleasure subscale; FLQ-WC = Weight concern subscale; FLQ-DHO = Diet–health orientation subscale; FLQ-DHL = Diet–health link subscale; TFEQ = Three-Factor Eating Questionnaire; TFEQ-CR = Cognitive restrictive behavior subscale; TFEQ-UE = Uncontrolled eating subscale; TFEQ-EE = Emotional eating subscale. * *p* < 0.01. ** *p* < 0.001.

## Data Availability

Data are available upon reasonable request.

## Supplementary Materials

The following supporting information can be downloaded at https://www.mdpi.com/article/10.3390/nu16193252/s1: Figure S1: Scree plot derived from parallel analysis of the exploratory factor analysis of the E-PID; Table S1: Verbal understanding and content validity of the E-PID Brazilian Portuguese version.

## Appendix A. Cross-Cultural Adaptation of the E-PID

### Appendix A.1. Cross-Cultural Adaptation of the E-PID

This study followed the guidelines for cross-cultural adaptation of health measurement instruments [17]. Initially, the first author of the scale was contacted and approved the development of the present study [8]. The EPID was translated from English (United States) to Portuguese (Brazil) by two independent translators (T1 and T2) and was forwarded to the research team for the development of the translation synthesis (ST). This synthesis was forwarded to two additional translators for the execution of a back-translation of the scale (RT1 and RT2) from Portuguese to English. All previous versions were discussed by a committee made up of in experts eating attitudes and behaviors and validation studies. The experts evaluated the E-PID’s semantic, cultural, conceptual, idiomatic, and operational equivalences, producing a pre-test version applied to a sample of 41 young women. The E-PID response options were adapted to a six-point Likert-type scale (0 = *I didn’t understand anything* to 5 = *I understood perfectly and I have no doubts*), which asked: “How much did you understand of what was asked in each question?” Furthermore, where respondents felt that the language was inappropriate, they were asked to justify the reasons and provide suggestions. Averages lower than three were considered inadequate for verbal comprehension [17,34].

### Appendix A.2. Results from the Cross-Cultural Adaptation

The original and translated items into Portuguese (Brazil) of the E-PID can be seen in Table S1. Certain adjustments were made to enhance comprehension among the target population. Specifically, in the response instructions of the instrument “disagree” (*discordo*) and “agree” (*concordo*), the qualifier “totally” (*totalmente*) was incorporated. Additionally, “neither disagree nor agree” (*nem discordo nem concordo*) was translated as “not disagree nor agree” (*não discordo nem concordo*). In item #2, the translation proposed “For food decisions, it makes sense to trust completely your instinct” (*Para decisões alimentares faz sentido confiar completamente no seu instinto*), which was modified to “For most food decisions, it makes sense to trust completely your instinct” (*Na maioria das decisões alimentares*, *faz sentido confiar totalmente no seu instinto*). In item #3, “I am a very intuitive eater” (*Eu sou um comedor muito intuitivo*) was rendered without “very” (*muito*) and with gender inflection applied to the words *um* (one), *comedor* (eater), and *intuitivo* (intuitive). Item #4, initially translated as “I usually think before making decisions related to food” (*Eu geralmente penso antes de tomar decisões relacionadas à alimentação*), was adjusted to “Before making food decisions, I usually think about them” (*Antes de tomar decisões alimentares*, *eu geralmente penso sobre elas*). Furthermore, in item #6, the translation of “I prefer to make plans regarding my eating behavior instead of leaving it to chance” (*Eu prefiro fazer planos em relação ao meu comportamento alimentar em vez de deixar isso ao acaso*) was revised to “I prefer to make plans regarding my eating behavior instead of leaving them to chance” (*Para decisões alimentares faz sentido confiar completamente no seu instinto*). Finally, a Content Validity Index (CVI > 0.80) was applied [34]. Good verbal understanding of the E-PID (*M* > 3) was obtained in the pre-test (*n* = 41), using a six-point Likert-type scale.
