# Supplementary material for: Psychometric Properties of the Preference for Intuition and Deliberation in Eating Decision-Making Scale among Brazilian Adult Women
Source: Nutrients. 2024 Sep 26;16(19):3252. doi: 10.3390/nu16193252 (PMC11478558; doi:10.3390/nu16193252)
Supplement: Supplementary file 1 [file nutrients-16-03252-s001.zip › nutrients-3183301-supplementary.pdf]

Supplementary Materials

Figure S1. Screeplot derived from parallel analysis of the exploratory factor analysis (EFA) of the Preference for Intuition and Deliberation in Eating Decision-making Scale (E-PID).

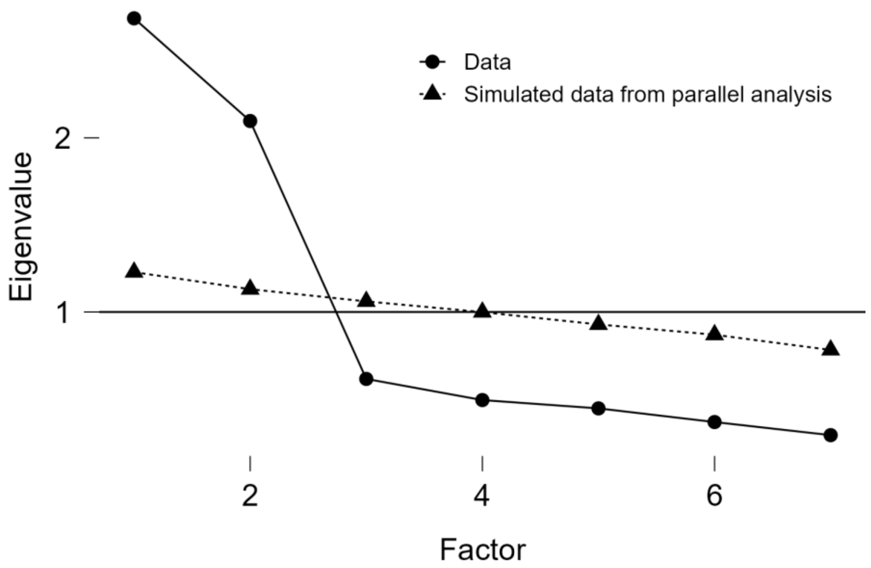

Table S1. Evaluation of verbal comprehension and content validity of the Brazilian Portuguese version of the Intuition and Deliberation in Eating Decision-making Scale (E-PID).

EPID items / *Brazilian Portuguese translation*		Verbal Comprehension Md (Mín-Max)	CVI
1)	When deciding what to eat, I rely on my gut feeling/ *Na hora de decidir o que comer, eu confio na minha intuição.*	5 (0-5)	1
2)	With most eating decisions, it makes sense to completely rely on your instinct/ *Na maioria das decisões alimentares, faz sentido confiar totalmente no seu instinto.*	4 (0-5)	0
3)	I am a very intuitive eater/ *Eu sou um(a) comedor(a) intuitivo(a).*	4 (0-5)	1
4)	Before I make eating decisions, I usually think about it/ *Antes de tomar decisões alimentares, eu geralmente penso sobre elas.*	4.5 (0-5)	1
5)	I think more about my plans and goals relating tomy eating behaviour than other people/ *Eu penso mais nos meus planos e objetivos relacionados ao meu comportamento alimentar do que outras pessoas.*	4 (0-5)	1
6)	I prefer making plans about my eating behaviour instead of leaving it to chance/ *Eu prefiro fazer planos em relação ao meu comportamento alimentar ao invés de deixá-los ao acaso.*	4.5 (0-5)	1
7)	I reflect on my eating behaviour/ *Eu reflito sobre o meu comportamento alimentar.*	5 (0-5)	1
	**Total**	4.4 (0-5)	0.85

Note: M_d = median; Min = minimum; Max = maximum; CVI = Content Validity Index.
